# The Importance of Genetic Testing for Familial Hypercholesterolemia: A Pediatric Pilot Study

**DOI:** 10.3390/medicina60101602

**Published:** 2024-09-29

**Authors:** Andreea Teodora Constantin, Corina Delia, Lucia Maria Roșu, Ioana Roșca, Ioana Streață, Anca-Lelia Riza, Ioan Gherghina

**Affiliations:** 1 Faculty of Medicine, University of Medicine and Pharmacy “Carol Davila”, 020021 Bucharest, Romania; andreea.constantin@drd.umfcd.ro (A.T.C.);; 2 Pediatrics Department, National Institute for Mother and Child Health “Alessandrescu-Rusescu”, 020395 Bucharest, Romania; 3 Faculty of Biology, University of Bucharest, 030018 Bucharest, Romania; 4 Faculty of Midwifery and Nursery, University of Medicine and Pharmacy “Carol Davila”, 020021 Bucharest, Romania; 5 Neonatology Department, Clinical Hospital of Obstetrics and Gynecology ”Prof. Dr. P. Sârbu”, 060251 Bucharest, Romania; 6 Genetics Department, University of Medicine and Pharmacy, 200349 Craiova, Romania; 7 Regional Center for Medical Genetics Dolj, 200642 Craiova, Romania

**Keywords:** familial hypercholesterolemia, cholesterol, pediatric

## Abstract

*Background and Objectives:* Familial hypercholesterolemia (FH) is a genetic disease that is massively underdiagnosed worldwide. Affected patients are at high risk of cardiovascular events at young ages. Early intervention in childhood could help prevent heart attacks and cerebral strokes in these patients. *Materials and Methods:* We conducted an interventional study including 10 patients that previously underwent genetic testing for familial hypercholesterolemia. These patients received lifestyle and diet recommendations that they followed for a year before being reevaluated. *Results:* Patients with negative genetic testing were able to achieve lower levels in their lipid panel values compared to the patients with positive genetic testing, with lifestyle changes alone. LDL-cholesterol levels decreased by 18.5% in patients without FH while patients genetically confirmed with FH failed to achieve lower LDL-cholesterol levels without medication. *Conclusions:* Genetic testing for FH is not always part of screening algorithms for FH. Some studies even advise against it. Our study proved the importance of genetic testing for FH when suspecting this disorder and choosing the treatment course for patients.

## 1. Introduction

Familial hypercholesterolemia (FH) is a genetic disease characterized by high LDL-cholesterol levels [1,2,3]. Each child of an affected individual has a 50% chance of inheriting the condition from birth [4]. Despite all the information we have now on FH, it is massively underdiagnosed [2]. In Europe, it is estimated that more than 2 million individuals with FH are currently undiagnosed [5]. Most pediatric patients with FH do not have remarkable symptoms and are being diagnosed as adults when cardiovascular events occur [6]. In a study from the U.S.A., approximately 2% of patients that had a heart attack at a young age had a mutation in the LDL-receptor gene (suggesting FH) [7].

Recent studies estimate the 1:313 individuals are affected by FH making it one of the most frequent genetic disorders in the world [8]. In Denmark, the prevalence of FH is estimated at 1:137 individuals [9]. Organizations such as European Atherosclerosis Society and the American Academy of Pediatrics recommend universal screening for FH in pediatric patients [10,11,12,13]. Most studies recommend screening for FH before adolescence. LDL-cholesterol levels suggestive for FH vary among authors from 140 mg/dL to 190 mg/dL [14,15,16,17]. Genetic testing for FH confirms the diagnosis but it is not gold standard. There are some studies against introducing genetic testing in FH diagnosis algorithms [18].

Most cases of FH are caused by mutations in the LDL-receptor (LDLR). A small number of cases (5%) are due to mutations in the LDL-receptor binding region of the APOB gene while gain-of-function PCSK9 (protein-subtilisin kexin 9) mutations account for 1 to 2% of cases [19].

A systematic review of randomized controlled nutritional studies on patients with FH suggests there is a lack of conclusive data on the effectiveness of diet manipulation for decreasing LDL-cholesterol levels in these patients [20]. Patients with FH often require pharmacological treatment. However, diet adds incremental health benefit for them [21].

## 2. Materials and Methods

We conducted an interventional study including 10 patients that were genetically tested for familial hypercholesterolemia. These patients were selected from a larger database that included pediatric patients with LDL-cholesterol over 130 mg/dL. Inclusion criteria were LDL-cholesterol over 130 mg/dL, no previous genetic testing for familial hypercholesterolemia and agreement to take part in the study.

Patients were clinically and paraclinically evaluated. Blood was drawn to perform genetic testing for familial hypercholesterolemia and fasting lipid panel evaluation. The results of the genetic testing for the patients included in this study were previously published [3]. According to their genetic testing results patients were divided into two groups (genetic-positive and genetic-negative).

Parents were questioned whether they have a family history of known relatives diagnosed with familial hypercholesterolemia or suggestive for early cardiovascular disease (events such as stroke or myocardial infarction in a first-degree relative less than 55 years old in men and less than 65 years old in women). Clinical examination included searching for clinical signs suggestive of FH such as xanthomas (especially tendon xanthomas). Weight, height and blood pressure were measured for each patient during their initial visit. For paraclinical evaluation, patients were instructed to have been fasting for at least 6 h. Lipid panel including total cholesterol, LDL-cholesterol, HDL-cholesterol, triglycerides, apolipoprotein A and apolipoprotein B were evaluated for each patient during the initial visit and during reevaluation.

All patients received at their initial visit recommendations for lifestyle changes and dietary management aiming to lower their LDL-cholesterol values. One year later they came for reevaluation. The recommendations for lifestyle changes and dietary management were written after consulting several guidelines [22,23,24,25] and adapted to local customs.

Each family received a sheet with all the dietary and lifestyle recommendations clearly stated. General recommendations included daily intake of a variety of nutrients from all food groups (dairy, eggs, meat, fruits and vegetables). It was recommended that all food and drinks contained as little as possible salt, sugar or sweeteners that add calories.

Fats consumption recommendations were grouped by the patients’ age. For the age group of 1 to 2 years we did not recommend any restriction in fat consumption. For the age group of 2 to 3 years a fats consumption of approximately 25 to 30% of daily energetic intake with less than 10% saturated fatty acids and as few as possible trans-unsaturated fatty acids was recommended. For the age group of 4 to 18 years (where most of our patients were situated) a fats consumption of approximately 25 to 35% of daily energetic intake with less than 10% saturated fatty acids and as few as possible trans-unsaturated fatty acids was recommended. The sheet included examples of food rich in saturated fats and food with trans fats that should be avoided as much as possible. The sheet received by our patients also contained advice on how to reduce their saturated fatty acids consumption (such as reading labels, choosing low-fat meat, low-fat dairy products, low-fat homemade snacks, cooking food on the grill, in the oven or by steaming rather than frying).

For fruit and vegetables, patients were advised to choose a “colorful” variety daily (at least 2 fruit servings and 3 vegetable servings). Fruits should be eaten whole, raw, boiled or roasted. Fruit juice should be limited to twice a week and to 120 mL to children aged from 1 to 3 years, 120–180 mL for children aged from 4 to 6 years and 240 mL for children older than 7 years.

Whole grain cereals were recommended. Cow milk consumption was recommended to be at least 480 mL/day for children aged from 1 to 2 years, 250–720 mL/day for children aged from 2 to 8 years and 720 mL/day for children over 9 years old. For children over 2 years old, 1.5% fat cow milk was recommended.

Liquids consumption was recommended to be around 1.5 to 2 L/day and included: water, unsweetened tea, lemonade and other unsweetened beverages.

Patients were advised to have at least 60 min per day of moderate physical activity (walking, hiking) or intense physical activity (running, biking, dance, martial arts, football, basketball etc.).

Parents were recommended to implement the changes as a family and not impose them on the child alone. This recommendation was meant to avoid the child feeling as if they were being punished and to increase adherence to the recommendations.

The study was approved by the ethics committee of the National Institute for Mother and Child Health “Alesssandrescu-Rusescu”, Bucharest, Romania under approval number 12747/16.07.2020 and was conducted respecting the Helsinki Declaration for Human Rights.

Descriptive and statistical analysis was performed using Epi Info™ For Windows, version 7.2. The parametrical or non-parametrical tests used considered the significance level threshold as 0.05.

## 3. Results

This study included 10 patients, four of whom were girls. All 10 patients were suspected to have familial hypercholesterolemia based on clinical criteria (LDL-cholesterol value, family history). The diagnosis was confirmed via genetic testing in six patients. They had mutations in LDLR, APOB and PCSK9 genes. The mutations identified in the six patients that were confirmed with familial hypercholesterolemia are presented in Table 1.

The mean age of patients included in this study was 9.9 (±3.6) years. Clinical data for the study group are presented in Table 2.

On clinical examination none of the patients included in this study showed any clinical signs associated with FH.

Body Mass Index (BMI) was calculated for each patient at the initial visit and percentiles were used according to age and sex. Three of the patients from the study group were underweight, one was overweight, three had class I obesity and one class III obesity. The underweight patients were all genetic-positive for FH while only the patient with class III obesity was in the genetic-negative group.

Lipid profile results for the study group at their initial visit are presented in Table 3. We noticed that patients in the genetic-positive group had slightly higher total cholesterol and LDL-cholesterol as well as higher Apolipoprotein A (Apo A) and Apolipoprotein B (Apo B values).

For the lipid panel results for the study group, the mean total cholesterol level in the study group was 233.1 (±37.0) mg/dL. The mean LDL-cholesterol level was 163.5 (±28.9) mg/dL. The mean HDL-cholesterol level in the study group was 59.0 (±15.6) mg/dL. The mean triglycerides level was 80.4 (±36.5) mg/dL. For the whole study group, after one year of lifestyle changes, total cholesterol levels decreased by 9.9% while LDL-cholesterol levels decreased by 8.5%.

**Figure 1:**
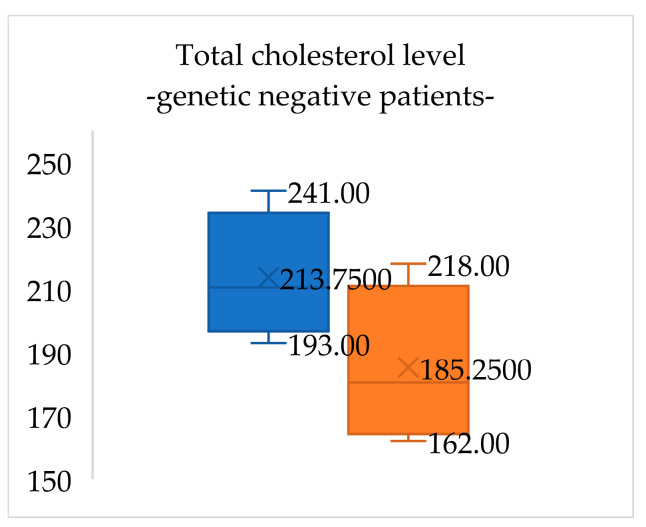
Total cholesterol levels in genetic-negative patients for FH at the initial evaluation (blue) and when reevaluated after one year of lifestyle management (orange). Total cholesterol levels in these patients decreased by 13.3%.

**Figure 2:**
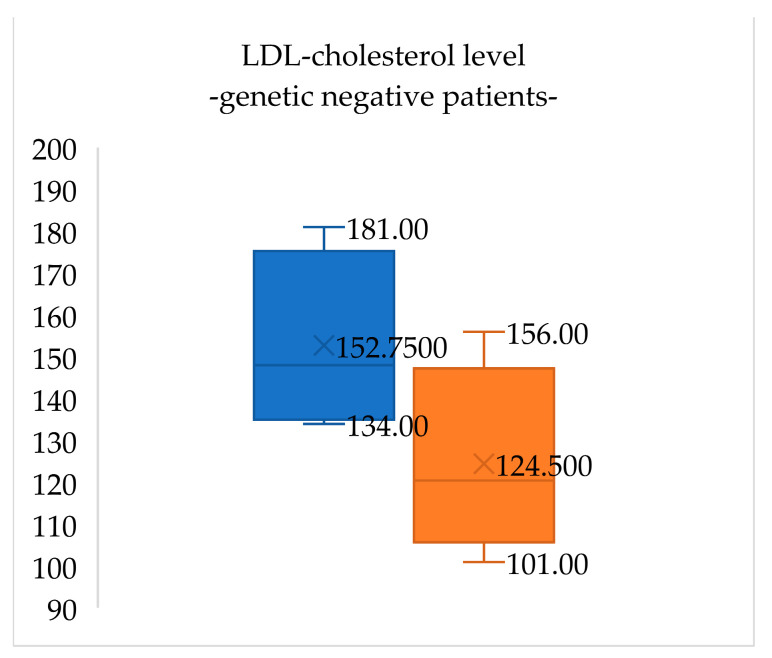
LDL-cholesterol levels in genetic-negative patients for FH at the initial evaluation (blue) and when reevaluated after one year of lifestyle management (orange). LDL-cholesterol levels in these patients decreased by 18.5%.

At the initial visit, lipid panel results for patients negative for FH (four patients) were total cholesterol 213.7 (±20.0) mg/dL, LDL-cholesterol 152.7 (±21.6) mg/dL, HDL-cholesterol 49.8 (±13.2) mg/dL and triglycerides 95.3 (±53.8) mg/dL. After one year of lifestyle changes, total cholesterol levels in these patients decreased by 13.3% and LDL-cholesterol levels decreased by 18.5% (Figure 1 and Figure 2).

**Figure 3:**
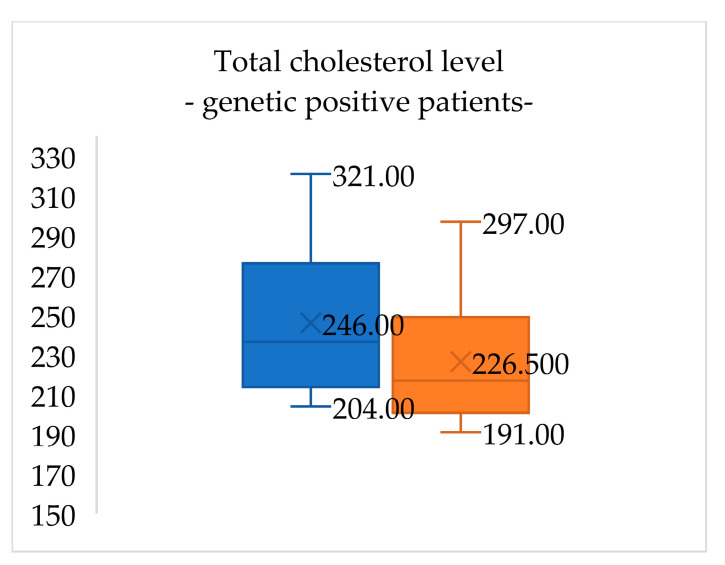
Total cholesterol levels in genetic-positive patients for FH at the initial evaluation (blue) and when reevaluated after one year of lifestyle management (orange). Total cholesterol levels in these patients decreased by 7.9%.

**Figure 4:**
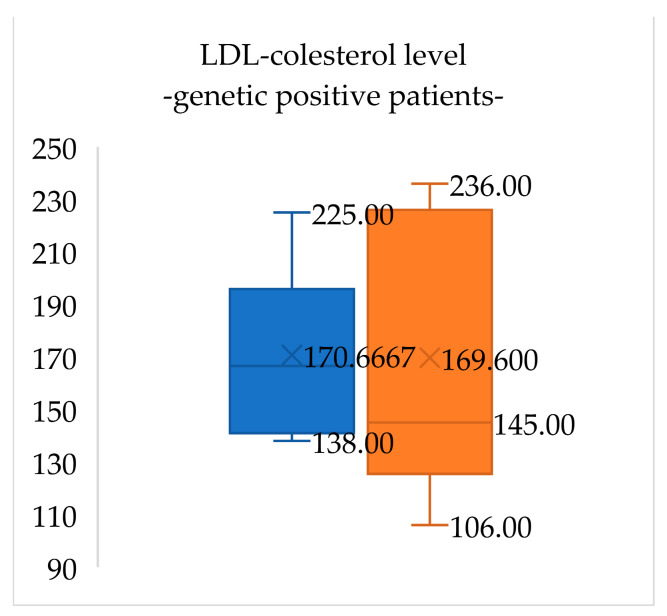
LDL-cholesterol levels in genetic-positive patients for FH at the initial evaluation (blue) and when reevaluated after one year of lifestyle management (orange). LDL-cholesterol levels insignificantly decreased in these patients (0.6%).

At the initial visit, lipid panel results for patients with confirmed FH (six patients) were total cholesterol 246.0 (±31.0) mg/dL, LDL-cholesterol 170.7 (±24.4) mg/dL, HDL-cholesterol 65.2 (±15.6) mg/dL and triglycerides 70.5 (±19.3) mg/dL. After one year of lifestyle changes, total cholesterol levels in these patients decreased by 7.9% and LDL-cholesterol levels decreased by 0.6% (Figure 3 and Figure 4).

Patients in the genetic-positive group had higher total cholesterol and LDL-cholesterol levels at the initial visit, compared with patients in the genetic-negative group. They also did not manage to lower their total cholesterol and LDL-cholesterol levels via igieno-dietetic treatment alone.

## 4. Discussion

FH is found in the general population in 1 in 300 people, 1 in 15 persons with premature coronary artery disease and 1 in 30 persons with coronary artery disease [26].

A recent study estimated that 47 years of opportunistic cholesterol screening and cascade genetic testing for familial hypercholesterolemia would detect only 25% of individuals with FH in the population [27].

Screening programs and registries for familial hypercholesterolemia (FH) have been implemented in some countries worldwide such as Vietnam, Austria, Czech Republic, Bulgaria, Italy, United Kingdom and others [28,29]. In Romania there is no screening program or registry for familial hypercholesterolemia.

There are several obstacles in implementing a screening program for FH that include genetic testing in our country as well as in many other European countries. Prices for testing an individual without known family mutation vary between EUR 300 and EUR 1000 in Scandinavian countries [30]. In the Netherlands, the price of DNA testing for FH is over EUR 2500 [31]. In our country, Romania, the cost of genetic testing for FH can reach EUR 1000 and higher.

One of the most active screening programs for FH was implemented in the Netherlands. It was funded by the Dutch Ministry of health, and it evolved from a regional pilot research project to full nationwide population screening with more than 64,000 subjects that had undergone genetic testing for FH [32]. During this program, at the time of the initial examination, only 39% of the adult patients included were receiving some form of cholesterol-lowering treatment. This percentage had risen to 93% one year later [32].

In a study conducted in the United States about one-third of clinically diagnosed patients with FH showed spontaneous interest in receiving free genetic testing. From those tested, 58% were positive for a FH gene [33]. A simulation analysis for the cost-effectiveness of cascade genetic testing for familial hypercholesterolemia in the United States demonstrated an average discounted cost per life year gained per person and associated confidence interval of less than USD 50,000 if testing was started before age 40 [34].

In the UK healthcare setting both cascade testing and child–parent cascade screening for FH have been assessed with estimative incremental cost effectiveness ratios of GBP 5806 per quality-adjusted life years (QALY) and 12,480/QALY, respectively [27].

Prognosis and complications in patients with FH include accelerated atherosclerosis and premature coronary heart disease [35]. The clinical outcome in these patients is related to the degree and duration of exposure to elevated LDL-cholesterol [36]. A study on Japanese patients found that patients with FH diagnosed during childhood had much better prognoses than those diagnosed during adulthood despite having higher LDL-cholesterol levels under milder lipid-lowering treatment [37]. Individuals with homozygous FH are at very high risk of death, on average at 18 years of age, but there have also been reports of death in childhood [35,38,39,40].

There are three primary genes associated with FH: LDLR, APOB and PCSK9 [41]. Patients diagnosed with FH usually have a heterozygous pathogenic variant in one of these three genes (heterozygous FH) and can rarely have biallelic pathogenic variants these genes (homozygous FH) with a more severe phenotype [41]. Individuals who are phenotype-positive and genotype-negative (such as the four genetic-testing-negative patients in our study) may have another genetic cause contributing to their raised LDL-cholesterol levels. Based on clinical criteria alone, in only 40–70% of patients a pathogenic variant in one of the three primary genes responsible for FH can be identified [41].

The inheritance pattern for FH has been described as autosomal semidominant since both alleles can contribute to the phenotype, additively raising the LDL-cholesterol level [42]. There are extremely rare situations where an autosomal recessive inheritance pattern is described in individuals with pathogenic variants of the LDLRAP1 gene [43,44]. FH has a 100% penetration rate and only a few cases with de novo pathogenic variants have been described [45]. When a patient is diagnosed with FH, we can identify relatives who are affected via cascade screening, however, genetic testing is not usually conducted for them because a high LDL-cholesterol level is sufficient to make a diagnosis [43].

Clinical features of heterozygous FH are rarely present in children while early-onset atherosclerosis can be observed in studies of the carotid intimal-media thickness [46]. In terms of clinical examination, three physical signs (tendon xanthomas, xanthelasma and corneal arcus) have been associated with heterozygous FH [47]. In our study, none of the children included showed any clinical signs of FH. Carotid intimal-media thickness was not evaluated.

There is no worldwide consensus regarding diagnostic criteria and LDL-cholesterol values that indicate FH. Several other variables such as family history of high LDL-cholesterol level, early atherosclerosis or diagnosed FH are taken into consideration [15,17,26,48,49]. A recent study carried out universal screening for FH in two populations and found that for LDL-cholesterol of 135.3 mg/dL, the overall sensitivity and specificity for confirming FH were 90.5% and 55.3%, respectively [5]. However, if only LDL-cholesterol levels are used for cascade screening, up to 20% of family members with a positive LDLR mutation but with LDL-cholesterol below the 90th percentile may go undiagnosed [4,50].

In our study, all underweight patients were genetic-positive for FH. This finding is consistent with other studies that report only 5% of patients with clinical FH diagnosis being obese, without statistically significant difference between genders [51]. In our country the main perception of the general population is that high cholesterol levels are associated with being overweight and obese. Therefore, since there is no screening program implemented, parents and sometimes even medical professionals tend to overlook the lipid panel evaluation in normal and underweight pediatric patients.

Lifestyle factors may play a key role in increasing the risk of cardiovascular disease (CAD) among carriers of a FH variant [52]. Carriers of a FH variant have an increased risk of CAD (three times higher), but it varies significantly according to lifestyle characteristics [52]. Current guidelines suggest that key pillars for clinical management of patients with familial hypercholesterolemia are a healthy diet pattern, regular activity and avoidance of tobacco while focusing on lowering of LDL-cholesterol [17,26,52,53,54]. In our study, all patients received recommendations for lifestyle changes, regardless of their genetic status.

Adherence to a healthy lifestyle pattern has been shown to be associated with reduced risk for cardiovascular disease regardless of their FH mutation status [52]. Despite this, LDL-cholesterol lowering effects of reducing saturated fatty acids intake is rather modest in patients with FH [55]. The results of our study were consistent with this affirmation since patients with identified mutations for FH were essentially not able to lower their LDL-cholesterol levels without medication. In what concerns pharmacotherapy for FH in children, the first line of treatment is considered to be statins, in some cases along with ezetimibe, starting as early as age 8 to 10 years old [23,56,57]. Their effect on LDL-cholesterol levels and reducing carotid-intima thickness (sometimes already present in children with FH) has the potential to reduce their risk of cardiovascular disease to the level of general population [58]. There is also a tendency to lower the age of patients that can start treatment with statins since patients with severe FH require pharmacotherapy early on [16,56,59]. For the treatment of patients with severe hypercholesterolemia, specific interventions were developed such as lomitapide (a small molecule inhibitor of microsomal triglyceride transfer protein) that was approved for the treatment of homozygous familial hypercholesterolemia in 2012 in North America and Europe [60]. Protein convertase subtilisin/kexin typer 9 (PCSK9) inhibitors, initially used for the treatment of homozygous FH, were recently studied for use in patients with heterozygous FH as well [61]. When combined with statins, PCSK9 inhibitors can help lower LDL-cholesterol levels by 50 to 60% [17,62,63].

Adolescent female patients that receive treatment with statins should be informed about the possible teratogenic effect and should have access to gynecological consultations. If oral contraception is prescribed for them, LDL-cholesterol and triglycerides levels might increase as a side effect. Ideally patients that are planning to become pregnant should stop statins treatment three months before conception, during pregnancy and during breastfeeding [64,65].

Target levels for LDL-cholesterol in patients with FH vary among societies and authors. The European Atherosclerosis Society pediatric guidelines recommend reduction by at least 50% for patients from eight years old and to less than 130 mg/dL for patients over ten years old [16]. The American College of Cardiology/American Heart Association guidelines specify that the acceptable LDL-cholesterol level in pediatric and adolescent patients is less than 110 mg/dL [23,48]. They also recommend starting treatment with statins from as young as eight years old [23,48]. In our study, the group of patients with negative genetic testing for FH achieved a mean LDL-cholesterol level close to the target value recommended by the European Atherosclerosis Society via diet and physical exercise alone. A recent study [66] on more than 400 adult patients with angiographically proven cardiovascular disease found low rates of longitudinal lipid target achievement, especially in those with FH. Another study from 2022 [52] reported that only 2.4% of their enrolled patients managed to achieve their recommended target of less than 100 mg/dL for LDL-cholesterol.

According to the Italian Society for the Study of Atherosclerosis (SISA), patients diagnosed with FH should undergo at least one visit a year, even if clinical signs of cardiovascular disease are absent [55]. They also recommend evaluation and follow-up by a clinical cardiologist in symptomatic patients with FH [55].

Optimizing treatment for children and adolescents diagnosed with FH requires a multidisciplinary medical team that includes the family doctor, the pediatrician and cardiologist [67]. Support groups have a significant role helping patients become emancipated, increasing risk awareness and informing on treatment options. These support groups can also improve communication between patients and medical professionals [68].

Transitioning from adolescence to adulthood is a delicate timeframe for patients with FH and for treatment adherence. In order to achieve long term compliance to treatment it is essential to start diet and lifestyle education in childhood [25].

Our study has certain limitations. The first one is the small number of participants which was due to the high cost of genetic testing for familial hypercholesterolemia and the falling-out of several patients after the initial visit. Another limitation is the fact that adherence to lifestyle changes was not evaluated.

In our study there were obvious differences between initial treatment response in patients with confirmed FH compared to those without, thus emphasizing the importance of confirming FH via genetic testing.

## 5. Conclusions

This study emphasizes the importance of genetic testing for patients with familial hypercholesterolemia and the importance of knowing if we are dealing with a FH variant carrier when approaching the management of these patients.

However, since genetic testing for FH is not available worldwide and the costs are high (especially in our country), recommendation of lifestyle changes should be offered universally to all patients suspected to have FH (not taking into consideration if they have undergone genetic testing or not). Diet treatment adds incremental health benefit to pharmacologic treatment in familial hypercholesterolemia [21].

Future studies on a regional and even national scale with more patients included are needed in order to validate our findings and emphasize the need for FH screening in or country. Also, studies on the cost-effectiveness of screening approaches in our healthcare environment would help implementing such a program.

## Figures and Tables

**Table 1:** Mutations identified in the six patients included in the genetic-positive group. These data were previously published [3].

Running Number	FH	Affected Gene	Variant		ACMG Variant Classification
1	Type 2 OMIM #144010 AD *	*APOB*	NM_000384.3:c.10580G>A rs5742904 (p.Arg3527Gln)	Heterozygous	Likely pathogenic
2	Type 1 OMIM #143890 AR */AD	*LDLR*	NM_000527.5:c.1618G>A rs769370816 (p.Ala540Thr)	Heterozygous	Pathogenic
3	Type 1 OMIM #143890 AD/AR	*LDLR*	NM_000527.5:c.502G>A rs200727689 (p.Asp168Asn)	Heterozygous	Pathogenic
4	Type 1 OMIM #143890 AD/AR	*LDLR*	NM_000527.5:c.81C>G rs2228671 (p.Cys27Trp)	Heterozygous	Likely pathogenic
5	Type 3 OMIM #603776 AD	*PCSK9*	NM_174936.4:c.836C>T rs1049662014 (p.Pro279Leu)	Heterozygous	VUS
6	Type 3 OMIM #603776 AD	*PCSK9*	NM_174936.4:c.836C>T rs1049662014 (p.Pro279Leu)	Heterozygous	VUS

* AD autosomal dominant; AR autosomal recessive.

**Table 2:** Clinical data for the study group, the genetic-positive group and the genetic-negative group. No statistically significant differences were identified.

	Study Group (n =10)	Negative Genetic Testing (n = 4)	Positive Genetic Testing (n = 6)
Gender	4 girls 6 boys	2 girls 2 boys	2 girls 4 boys
Positive family history	4 cases	3 cases	1 cases
Mean age	9.9 years (±3.6)	10.5 years (±4.7)	9.5 years (±3.1)
BMI	22.0 (±8.8)	26.0 (±9.7)	18.0 (±6.6)
Mean systolic blood pressure	115.8 mmHg (±13.3)	130.0 mmHg (±2.6)	108.7 mmHg (±13.9)
Mean diastolic blood pressure	65.2 mmHg (±5.2)	68.7 mmHg (±6.1)	63.3 mmHg (±4.1)

**Table 3:** Lipid profile results for the study group, the genetic-positive group and the genetic-negative group at the initial visit. No statistically significant differences were identified.

	Study Group (n = 10)	Negative Genetic Testing (n = 4)	Positive Genetic Testing (n = 6)	*p*
Total cholesterol	233.1 mg/dL (±37.0)	213.8 mg/dL (±20.1)	246.0 mg/dL (±41.6)	0.20
LDL-cholesterol	163.5 mg/dL (±28.9)	152.8 mg/dL (±21.6)	170.7 mg/dL (±32.8)	0.33
HDL-cholesterol	59.0 mg/dL (±16.0)	49.8 mg/dL (±13.2)	65.2 mg/dL (±25.6)	0.13
ApoA	146.9 mg/dL (±31.4)	115.0 mg/dL (±14.1)	157.5 mg/dL (±28.3)	0.09
ApoB	122.3 mg/dL (±20.0)	114.5 mg/dL (±0.7)	124.8 mg/dL (±22.9)	0.50

## Data Availability

Data are contained within the article.
